# Molecular Epidemiology of HIV-1 Subtype G in the Russian Federation

**DOI:** 10.3390/v11040348

**Published:** 2019-04-16

**Authors:** Anastasia Murzakova, Dmitry Kireev, Pavel Baryshev, Alexey Lopatukhin, Ekaterina Serova, Andrey Shemshura, Sergey Saukhat, Dmitry Kolpakov, Anna Matuzkova, Alexander Suladze, Marina Nosik, Vladimir Eremin, German Shipulin, Vadim Pokrovsky

**Affiliations:** 1 Central Research Institute of Epidemiology, 111123 Moscow, Russia; dmitkireev@gmail.com (D.K.); pavel.fj@yandex.ru (P.B.); a.lopatukhin@gmail.com (A.L.); pokrovsky.vad@yandex.ru (V.P.); 2 Skolkovo Institute of Science and Technology, 121205 Moscow, Russia; ekaterina.serova@skolkovotech.ru; 3 Clinical Center of HIV/AIDS of the Ministry of Health of Krasnodar Region, 350015 Krasnodar, Russia; shemsh@mail.ru; 4 Department of Epidemiology, Rostov State Medical University, 344022 Rostov-on-Don, Russia; sauhat@yandex.ru; 5 Rostov Research Institute of Microbiology and Parasitology, 344000 Rostov-on-Don, Russia; dimakolpakov@mail.ru (D.K.); matuzkova@yandex.ru (A.M.); sualrostov@mail.ru (A.S.); 6 Ilya Ilyich Mechnikov Research Institute for Vaccines and Sera, 105064 Moscow, Russia; mnossik@yandex.ru; 7 Republican Research and Practical Center for Epidemiology and Microbiology, 220114 Minsk, Belarus; eremin.vf@gmail.com; 8 Center of Strategical Planning and Management of Biomedical Health Risks of the Ministry of Health, 119121 Moscow, Russia; shipgerman@gmail.com; 9 Department of infectious diseases with courses of epidemiology and phthisiology, RUDN University, 117198 Moscow, Russia

**Keywords:** HIV-1, subtype G, molecular epidemiology, near full-length genome sequencing, unique recombinant forms, nosocomial outbreak, Elista

## Abstract

Although HIV-1 subtype A has predominated in Russia since the end of the 20th century, other viral variants also circulate in this country. The dramatic outbreak of HIV-1 subtype G in 1988-1990 represents the origin of this variant spreading in Russia. However, full genome sequencing of the nosocomial viral variant and an analysis of the current circulating variants have not been conducted. We performed near full-length genome sequencing and phylogenetic and recombination analyses of 11 samples; the samples were determined to be subtype G based on an analysis of the *pol* region. Three samples were reliably obtained from patients infected during the nosocomial outbreak. The other 8 samples were obtained from patients who were diagnosed in 2010–2015. Phylogenetic analysis confirmed that a man from the Democratic Republic of the Congo was the origin of the outbreak. We also found that currently circulating viral variants that were genotyped as subtype G according to their *pol* region are in fact unique recombinant forms. These recombinant forms are similar to the BG-recombinants from Western Europe, particularly Spain and Portugal. The limitations of subtyping based on the *pol* region suggest that these viral variants are more widespread in Europe than is currently supposed.

## 1. Introduction

The HIV pandemic remains a major global health problem with 36.9 million people living with HIV and 1.8 million people newly infected in 2017 [1]. One of the most important factors in the worldwide spread of HIV is its enormous genetic variability and rapid evolution [2]. 

Group M, which is responsible for most cases of infection globally, has been classified into nine subtypes (A-D, F-H, J and K) and vast numbers of recombinant forms (circulating and unique). The number of CRFs (circulating recombinant form) and URFs (unique recombinant form) is continuously increasing, with 97 CRFs detected to date. Recombination, which involves shuttling mutations between viral genomes and leads to major antigenic shifts or alterations in virulence, is a key reason for the high variability of HIV [3].

At the global level, the proportion of subtype B has increased, subtypes A and D have remained stable and subtypes C, G and CRF02_AG have decreased over the last 15 years. CRF01_AE, other CRFs and URFs have increased, leading to a consistent increase in the global proportion of recombinants over time [2,4,5,6]. 

In Eastern Europe and Central Asia, more than 50% of infections were caused by subtype A with notable contributions by subtype B and CRFs: the proportion of subtype A declined from 91.3% in 2005–2009 to 52.8% in 2010–2015, with a concomitant increase in the prevalence of subtypes B, C and CRFs [3,5,7].

Subtype A continues to predominate in the Russian Federation. However, the percentage of non-A infections has increased in recent years. Cases of infections caused by subtype A decreased from 91.75% in 2000–2001 to 70.55% in 2014–2015. The subtype B virus (7.90%) is the most commonly detected non-A variant, followed by the AG-recombinant with approximately 7.01% of cases, subtype G (1.3%) and subtype C (1.06%) [8].

The dramatic outbreak of HIV-1 subtype G in 1988-1990 resulted from an origin of this variant spreading in Russia during the early days of the HIV epidemic in the country. This first outbreak in the south of the former USSR in Elista has been the largest and most studied amongst the outbreaks that have occurred. Data resulting from epidemiological studies have revealed that between 1988 and 1990, more than 260 patients (mainly children) were infected at 17 healthcare facilities [9,10]. 

The study showed that HIV infection occurred because of violations of rules regarding the use and sterilization of medical instruments. Initially, the screening studies in Elista in 1988 detected two HIV-infected patients: a female blood donor and a young child who were examined due to clinical indications. An evaluation of their medical history revealed that the infected child and the female donor with her child had received medical care in the summer of 1988 at the same unit of a children’s hospital. An evident connection of the HIV-infected patients caused to suppose the presence of a nosocomial infection. Immediately after the establishment of this fact, active screening of all children who were in this unit at that time was conducted. Consequently, three infected children were found. Because of the probability of the long existence of a transmissible case cluster, an examination of all the children who had attended the hospital together with the discovered infected children, their parents and persons who had community-acquired and medical contacts was conducted. Parenteral and vertical routes of viral transmission were confirmed. HIV-1 subtype G was found in sequenced samples, which provided additional support that the original source of infection was a particular person. Data from the medical charts and carrying polls were used to determine that the original source of the outbreak was a man who had been in the Democratic Republic of the Congo for a long time. On his return to the USSR, he infected his wife and she had then transmitted HIV-1 to her new-born. This child with his mother became the first link in the chain of viral expansion in the hospital [9,10].

This outbreak was thoroughly investigated from an epidemiological point of view. However, there is a lack of information about the subsequent distribution of this variant throughout the country since that time. 

HIV-1 subtype G is rarely detected in the territory of Russia and the countries of the former USSR [11] and the prevalence of this subtype is not widespread in those areas today [8]. However, worldwide, subtype G is the sixth most prevalent HIV-1 clade and accounts for nearly 5% of all global infections. This subtype reaches its highest prevalence in some African countries, comprising 5–50% of HIV-1 infections [12]. Some subtype G strains have also disseminated out of the African continent. The most remarkable example is Portugal, where subtype G is the second most prevalent HIV-1 clade (>10%), after subtype B (>40%) [12,13,14]. In addition to Portugal, Spain has experienced an enormous spread of HIV; their epidemics are characterized by substantial viral diversity and distinct molecular properties compared to other European countries [15,16,17]. Non-B subtypes, particularly subtype G, were also very prevalent, with subtype G found in approximately 30% of all diagnoses [7].

The high prevalence of subtypes B and G in Portugal and Spain has also promoted the appearance of different types of BG-recombinant strains, including one circulating recombinant form (CRF14_BG) that was initially identified in Galicia, Northern Spain [2,16]. 

The study of minor viral variants circulating in different regions allows us to better characterize the epidemic, determine the origin and direction of the distribution of these variants and assess the interconnection of epidemics in different countries.

Therefore, the aim of the present work was to conduct near full-length genome sequencing of the samples that were determined to be subtype G based on an analysis of the *pol* region and to study the molecular epidemiology of HIV-1 subtype G in the Russian Federation.

## 2. Materials and Methods

Near full-length genome sequencing was performed for 11 clinical samples that were genotyped as subtype G according to their *pol* region. Three samples were obtained from patients from the outbreak of 1988–1990: a sample of blood lymphocytes from infected child Sh., year of sampling: 1989 (year of first positive immunoblot test: 1989) and two plasma samples of infected child S., years of sampling: 2012 and 2014 (year of first positive immunoblot test: 1990). Additionally, 8 plasma samples were obtained from patients who experienced antiretroviral therapy failure (years of first positive immunoblot test: 2010–2015). This study was approved by the Ethics Review Committee of the Central Research Institute of Epidemiology (Moscow, Russia).

The “RIBO-sol-E” kit (Central Research Institute of Epidemiology, Russia) was used for RNA extraction from the plasma samples and the “RIBO-prep” kit (Central Research Institute of Epidemiology, Russia) was used for DNA extraction from the blood lymphocytes.

The two-step protocol of receiving specific fragments of HIV-1 DNA was used for the amplifications [18]. In the first step, amplification combined with reverse transcription was performed using the SuperScript III One-Step RT-PCR System with Platinum Taq High Fidelity (cat. #12574-035, Invitrogen, Carlsbad, CA, USA). Amplification was performed according to the following protocol: 50 °C, 30 min; 94 °C, 2 min; 30 cycles of 94 °C, 15 s, 50 °C, 30 s and 68 °C, 3 min 30 s; and 68 °C, 7 min. The presence or absence of the reverse transcription stage was the only difference in the protocols when using different types of nucleic acids. Nested PCR was performed during the second stage using Q5 High-Fidelity DNA Polymerase (cat. #M0491S, New England Biolabs, Ipswich, MA, USA). Amplification was conducted according to the following protocol: 98 °C—30 s; 30 cycles of 98 °C, 10 s, 57 °C, 30 s and 72 °C, 2 min; and 72 °C, 5 min. The amplification scheme produces four overlapping DNA fragments of 2637 to 2927 base pairs. The total length of the amplified region was 8997 nucleotides according to reference strain HIV-1 HXB-2 (positions 626-9623, GenBank: K03455). The primers for the first and second amplification steps were described previously [19,20] or developed independently.

Amplified HIV-1 genome fragments were purified from the reaction mixture with magnetic particles called Sera-Mag Magnetic Speed Beads (Dia.: 1 µm, 3 EDAC/PA5, GE Healthcare Biosciences) prepared as described in Reference [21]. The nucleic acid concentrations in the fragments were measured using a Nano Drop 2000c spectrophotometer (Thermo Scientific, Waltham, MA, USA) and Qubit 2.0 fluorimeter (Invitrogen, Carlsbad, CA, USA).

Purified preparations of the amplified HIV-1 fragments were mixed in equal proportions and 50 ng of the final mixture was used to produce libraries for sequencing on an Illumina platform. The libraries were prepared according to the Nextera protocol (Illumina, San Diego, CA, USA) with the following correction: amplification was performed with Q5 High-Fidelity DNA Polymerase in the presence of the intercalating dye EvaGreen (Biotium, Fremont, CA, USA).

The quality and validity of the ratio sizes for the fragments of the libraries were assessed using an Agilent Bioanalyser 2100 (Agilent Technologies, Santa Clara, CA, USA). The libraries were 400–500 base pairs in size. Sequencing was performed on a MiSeq (Illumina, San Diego, CA, USA) with a MiSeq Reagent Kit V2 (500 cycles, cat #MS 102-2003).

The processing of the genome sequencing results, genome assembly and read mapping were performed using the Trimmomatic program [22] and an in-house developed variable viral genome assembler [23]. A consensus nucleotide sequence with a sensitivity threshold to minor populations of 20% was automatically created.

The sequences were deposited in GenBank under accession numbers MF614605-MF614615. The HIV-1 *pol* and near full-length genome sequence subtyping was performed using the Rega HIV-1 Subtyping Tool 3.0 [24]. Precise breakpoint locations were analysed with jpHMM (jumping profile Hidden Markov Model) software [25].

The database from Los Alamos [26] and the Russian national database [27] were the sources of the HIV-1 subtype G nucleotide sequences and the epidemiological data (gender, route of transmission, country of collection, date of first positive immune blot test and date of collection). 

Sixty-three nucleotide sequences from the Los Alamos database were selected for the near full-length genome tree (accessed on 16 January 2018). These sequences did not include the sequences from Russia. In addition, this batch was expanded up to 106 samples with the Los Alamos database and the Russian national database for the analysis of the *pol* region (accessed on 16 January 2018). All sequences were aligned using Muscle [28]. The alignment quality was manually checked in BioEdit [29].

Phylogenetic trees were estimated from the underlying nucleotide sequences using the approximate maximum likelihood (ML) method with bootstrap evaluation under the generalized time reversible (GTR+cat) model as a nucleotide substitution model—including a Γ distributed rate of heterogeneity amongst sites—as implemented in RaxML [30]. Further phylogenetic analysis was performed in FastTree v2.1 [31] to verify our results. Tree visualization and annotation was performed using FigTree v1.4 [32].

The phylodynamic analysis was conducted using a Bayesian approach as implemented in BEAST v1.8 [33]. We analysed sequences found within the Russian samples by using the GTR as the nucleotide substitution model with gamma heterogeneity and an uncorrelated relaxed clock model with lognormal distribution. The Markov chain Monte Carlo (MCMC) analysis was run for 10x106 generations and sampled every 1000 steps with the first 10% of samples being discarded as burn-in. The MCMC convergence and effective sample sizes (ESS) were checked using Tracer v1.5. A consensus tree was built and the distribution was assessed from the posterior tree using TreeAnnotator v1.8 23.

## 3. Results

### 3.1. Phylogenetic Analysis of Near Full-Length Genome Sequences of HIV-1 Subtype G

The near full-length genome sequencing revealed 11 HIV-1 nucleotide sequences (GenBank, MF614605-MF614615). Three of those sequences (MF614605-MF614607) were isolated from two patients who were infected during the nosocomial outbreak in Elista (USSR) from 1988–1990. The other 8 sequences (MF614608-MF614615) were isolated from patients whose first immunoblot and blood samples were obtained between 2010 and 2015. There were no epidemiological links between these 8 patients according to the epidemiological investigations (Table 1).

All samples were positive for HIV-1 subtype G according to *pol* region subtyping.

The phylogenetic tree was inferred by the ML method. Sixty-three sequences from other countries from the Los Alamos database were combined with the 11 sequences previously described. The final length of the analysed fragment was 8616 bp (according to HXB2, positions 796-9412).

**Figure 1:**
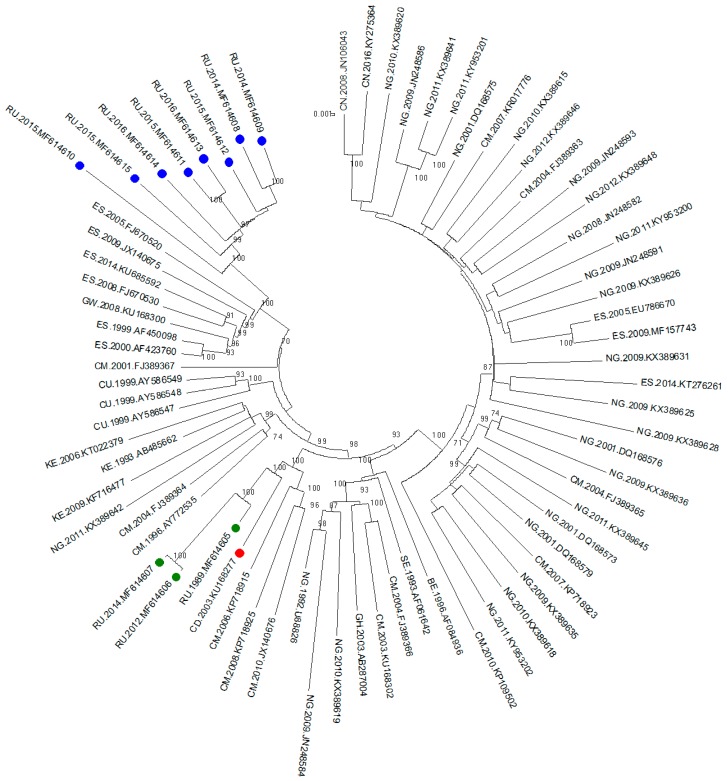
ML tree (500 bootstrap replicates) of HIV-1 subtype G sequences with a length of more than 8000 bp. The green circles indicate the sequences from the patients of the Russian nosocomial outbreak. The red circle indicates the sequence from the Democratic Republic of the Congo. The blue circles indicate the sequences of the infected patients from Russia who were diagnosed after 2010. The name of each sequence combines the country code, sampling year and accession number separated by dots. Only bootstrap values of more than 70% are shown.

The phylogenetic tree is shown in Figure 1. Three samples from the outbreak and sequences from the Democratic Republic of the Congo and Cameroon were clustered with high bootstrap support (100%). Interestingly, all the samples from Russian infected patients diagnosed after 2010 formed a well-defined monophyletic group with relatives from the samples from southern parts of the European Union (Spain) with high bootstrap support (100%).

Because the largest number of samples in the databases were represented by a fragment of the *pol* region (protease gene and a fragment of the reverse transcriptase gene), the next phylogenetic analysis was performed using this region.

The sequences of the *pol* region from the Los Alamos database and the Russian national database were combined with the near full-length genome sequences mentioned earlier. Finally, 117 sequences were selected for the phylogenetic analysis. After aligning and cutting the fragments at the edges, the analysed fragments were 1469 bp in length (according to HXB2, positions 2085–3554). A phylogenetic analysis was performed using the ML method.

**Figure 2:**
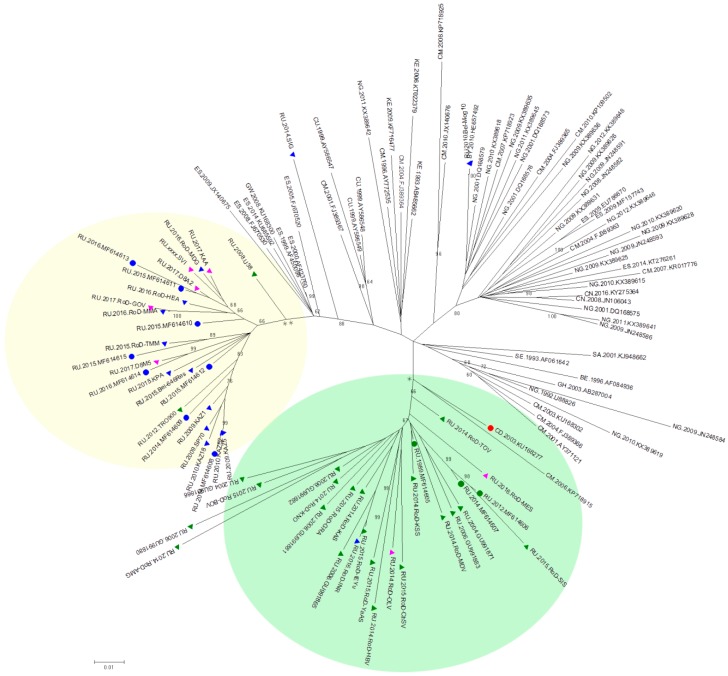
Maximum likelihood (ML) tree (500 bootstrap replicates) of the *pol* region sequences of subtype G. The red circle indicates the sequence from the Democratic Republic of the Congo. The green circles indicate sequences from patients from the nosocomial outbreak. The green triangles indicate sequences from Russian patients diagnosed between 1988 and 2006. The blue circles indicate the near full-length genome sequences of the infected patients from Russia who were diagnosed after 2010. The blue triangles indicate sequences from Russian patients diagnosed between 2007 and 2015. The purple triangles indicate sequences from Russian patients with unknown diagnosis dates. The sequence from the Democratic Republic of the Congo and samples from patients living in the territory covered by the outbreak are highlighted in the green oval. Samples from patients with a first positive immune blot test predominantly after 2007 are highlighted in the yellow oval. The name of each sequence consists of the country code, sampling year and accession number (Los Alamos) or sample name (Russian database) separated by dots. The symbol XXXX marks the samples for which the date of sampling is unknown. Only bootstrap values of more than 60% are shown. *-38% bootstrap support, **-41% bootstrap support.

The phylogenetic tree is shown in Figure 2. Similar to the first phylogenetic tree of near full-length genomes, 3 samples from the nosocomial outbreak are clustered with a sample from the Congo with well-supported topology (66%). In addition, 16 samples from the Russian database that were obtained from patients living in the territory covered by this outbreak were included in this cluster.

It should be noted that the remaining 8 samples for which near full-length genome sequencing was performed still form a separate cluster, which also includes 18 samples from the Russian database. Most of these samples were obtained from patients with a first positive immune blot test after 2007 but the region of collection was unknown.

### 3.2. Recombination Analysis of Near Full-Length Genome Sequences of HIV-1 Subtype G

According to the phylogenetic analysis, 8 of the 11 genomes form a separate cluster on the tree of near full-length genomes. This may have occurred as a consequence of the difference in the structure of the virus genome, which occurred during recombination. Therefore, a recombination analysis of these 8 samples was performed.

**Figure 3:**
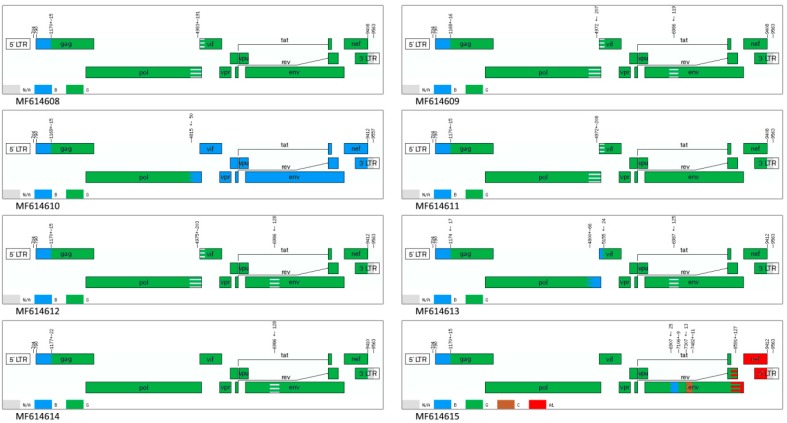
Scheme of the location of recombination patterns according to the jpHMM (jumping profile Hidden Markov Model) results. The patterns of homology are shown as follows: the G subtype is highlighted in green, the B subtype is highlighted in blue, the C subtype is highlighted in brown, the A1 subtype is highlighted in red and the unknown subtype (uncertain regions) is highlighted in grey.

Identifying possible recombination breakpoints using the jpHMM program showed that 7 of 8 samples were recombinant viruses of subtypes G and B, whereas the genome of sample MF614615 further contains inserts of subtypes A and C. Six genomes of 7 BG recombinants contain areas of uncertainty in the *pol/vif* and *env* genes. Instead of containing an uncertain region at the end of *pol* gene, sample MF614613 has an insert of subtype B; in sample MF614614, this respective fragment belongs to subtype G. The nucleotide sequence of the MF614610 genome from position 4831 (coordinates according to HXB2) contains a fragment that belongs to subtype B (Figure 3).

**Figure 4:**
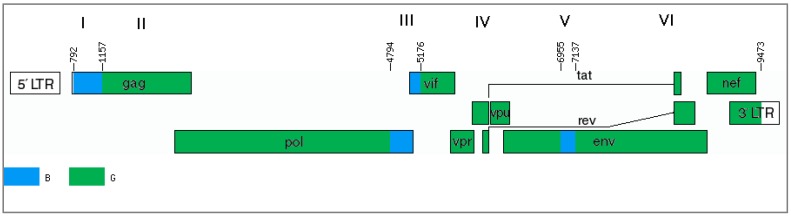
The mosaic structure of the new BG-recombinant form. The regions are numbered according to the jpHMM program. Areas III and V are subtype B or uncertain regions. The patterns of homology are shown as follows: the G subtype is highlighted in green and the B subtype is highlighted in blue.

The results of only one program (jpHMM) cannot provide sufficient evidence of the mosaic genome structure. In this case, it is advisable to use other methods, particularly the phylogenetic method. The genome of the studied recombinants can be divided into several regions and presented as alternations of genome fragments related to subtype B or uncertain regions (regions I, III and V), between which there are fragments related to subtype G (regions II, IV, VI) (Figure 4).

**Figure 5:**
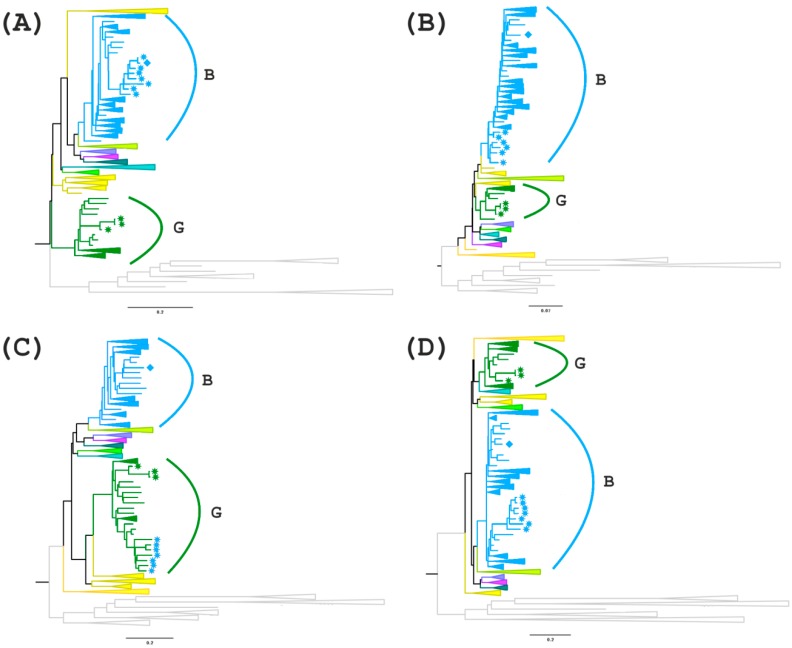
Phylogenetic trees for the nucleotide sequences of recombination regions I (**A**), III (**B**), IV (**C**) and V (**D**) of 11 genomes of subtypes G and BG. The B subtype is highlighted in blue and the G subtype is highlighted in green. BG recombinants are indicated with blue stars, except for MF614610. Sample MF614610 is indicated by a blue rhomb and the samples of subtype G from the outbreak are indicated by green stars.

For further analyses, regions I (*gag* gene fragment homologous to subtype B), III and V (uncertain regions in the *pol/vif* and *env* genes) were selected and phylogenetic trees were constructed (Figure 5A–D). In addition, the region between these areas with uncertain significance (region IV) is of interest since there is a possible formation of a tertiary recombinant between the new BG recombinant and subtype B in the of genome MF614610, which has an extended fragment of subtype B.

On the first tree (region I), all recombinant genomes form a separate branch that is related to genomes from the USA and Colombia. A more interesting picture is observed on the second tree for region III. MF614610 branches apart from the other recombinants from Russia and has a high similarity score compared to genomes from South America and Australia. The remaining 7 genomes cluster with subtype B but branch apart from the other samples. The phylogenetic tree of region IV confirms the presence of a G subtype region in the 7 recombinant genomes and a B subtype region in the genome of MF614610, which form one clade with the genomes from Europe, particularly from Ukraine and Germany. The analysis of region V showed that although all 8 recombinant genomes belong to subtype B, 7 of them form a group with the genomes from North and South America and Australia. MF614610 occupies, as in the previous case, a separate position and is similar to the genomes from Eastern Europe (Russia and Ukraine) (Figure 5).

### 3.3. Estimation of tMRCA of Subtype G Samples and New BG-Recombinant Forms

A molecular clock analysis allows us to determine the possible time appearance of a new BG recombinant. A possible location for the emergence of a new recombinant is the western part of the European Union, so the sample should include samples from this region. This analysis was conducted on a fragment of the *pol* gene, which belongs to subtype G.

On a Bayesian phylogenetic tree, subtype G samples from the outbreak of nosocomial infection in southern Russia in Elista form a monophyletic clade. A possible ancestor is from the countries of Central or West Africa (Cameroon, Kenya) and dates back to 1979 [95% HPD (highest posterior density): 1968–1983]. Notably, ancestors of this sample and samples of the new BG- recombinant form distinct monophyletic branches. The date of separation can be considered 1975 [95% HPD: 1961–1984].

**Figure 6:**
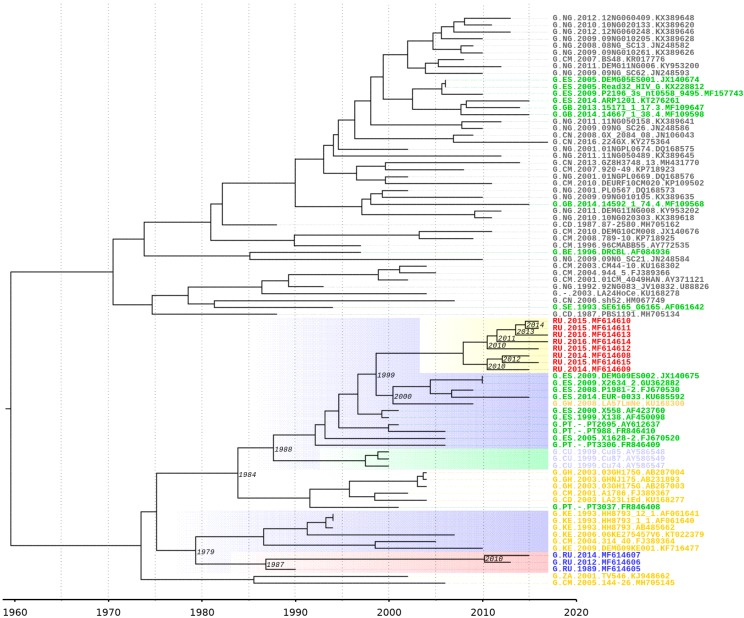
Dated Bayesian phylogenetic tree of subtype G and new BG recombinants. The BG recombinants are highlighted in red, the G subtype from patients from the nosocomial outbreak is highlighted in blue, samples from Europe are highlighted in green and samples from Africa are highlighted in yellow and black. The branch from Elista outbreak samples is shown in red and the branch from the BG recombinants is shown in yellow. The horizontal scale axis at the bottom corresponds to the calendar year. Nodes of branches contain the average 95% highest posterior density (HPD).

According to an analysis on the spread of the HIV-1 from Africa to Europe, a centre of infection has emerged in the south of Europe and includes Spain and Portugal [12,34]. The clustering of 8 Russian recombinant genomes with those from Spain and Portugal can date back to 1984 [95% HPD: 1971–1992] and neighbours adjacent to this branch from Cuba can date back to 1988 [95% HPD: 1977–1993]. All of these genomes have a common precursor from Africa. The tMRCA of the ancestral node of the BG cluster and European samples was estimated to be 1999 [95% HPD: 1995–2003] (Figure 6).

## 4. Discussion

The HIV-1 pandemic is not uniform; instead, it is complex and dynamic. The spread and evolution of HIV have caused various distributions of subtypes, CRFs and URFs worldwide; these distributions change over time in countries, regions and globally. It is essential to continuously monitor the diversity and spread of HIV-1 worldwide as the pandemic matures.

In recent years, the epidemic has evolved with an increase in the prevalence of non-B subtypes and recombinants in Western and Central Europe. In some countries, non-B clades have spread amongst the native population, such as subtype G in Portugal. Likewise, subtype G has been the dominant subtype in Nigeria and throughout West Africa over the past decade [14]. Globally, subtype G accounted for nearly 5% of people living with HIV of all HIV-1 infections in 2010–2015.

Subtype G, which also circulates in Spain, has recombined with subtype B, which likely occurred in Portugal early in the history of the epidemic. This resulted in CRF14_BG, which has been detected in Portugal, at a low prevalence in the region of Galicia, Spain and in some other European regions [7].

By contrast, the HIV-1 epidemic in former Soviet Union countries remains more homogeneous with the A_FSU_ variant that predominates throughout the USSR. In the last 15 years, there has been an increase in heterogeneity of the viral population, which is expressed as an increase in the proportion of non-A subtypes and recombinant forms in Russia. HIV-1 subtype G is rarely detected throughout the territory of this country but the circulation of subtype G viruses of the Iberian variant in Russia and neighbour countries has already been described [35]. It should be noted that HIV-1 subtype G caused the first outbreak in 1988-1990 in the territory of the former USSR. However, a full genome sequencing of the nosocomial viral variant and analysis of the current circulating variants have not been conducted.

We performed an analysis of subtype G in the Russian Federation. Three samples from two patients from the outbreak were obtained. One sample had a sampling date of 1989 and was collected soon after the patient was infected. Two other samples with sampling dates from 2012 and 2014 were collected from the second patient. In our work, near full-length genome sequencing of these samples was conducted. Three sequences (MF614605, MF614606 and MF614607) were classified as subtype G according to the subtyping results of the Rega HIV-1 Subtyping Tool 3.0. The results of the phylogenetic analysis demonstrated that these sequences cluster jointly, which confirms the origin of the viruses isolated from two patients as derivates of one ancestor. Based on the results of the phylogenetic analysis, one may conclude the genetic similarity of this virus to the Congo virus, which agrees with the epidemiological data. According to the literature [9], the primary cause of infection was a man who had lived in the Democratic Republic of the Congo for a long time. The nosocomial outbreak occurred after his return to Russia. The formation of an apparent cluster with a single sample from the Congo as an ancestor confirms the origin of the imported HIV-1 variant from this region.

We also performed near full-length genome sequencing of 8 samples of HIV-1 that were classified as subtype G according to the results of the *pol* region subtyping. These samples were obtained from patients with HIV infection that was confirmed between 2010 and 2015. The results of the phylogenetic analysis of the near full-length genome sequences showed that these samples are clustered with European samples and are significantly separate from the outbreak samples.

An analysis of the 8 genomes showed that they have a mosaic structure and are the result of recombination between subtypes G and B. Most of the genome belongs to subtype G; at the beginning there is an extended region with similarity to subtype B. In addition, there are two areas in which programs cannot reliably determine the presence/absence of homology with subtype B. Such results may be caused by the structure of the area. Further phylogenetic analysis confirmed the presence of subtype B regions in the *pol/vif* and *env* genes (III and V recombination regions), which may indicate the emergence of a new circulating recombinant form. Recombination breakpoints of the new form differ from known BG recombinants that are registered in the GenBank database. Genomes of samples MF614610 and MF614615 have different structures. MF614610 contains one large region from the end of the *pol* gene to the end of the whole genome (coordinates from 4794 according to HXB2); likewise, MF614615 additionally contains parts similar to subtypes C and A. The MF614610 sample is also assumed to have additional recombination between the new BG-recombinant form and subtype B. The almost complete coincidence of the recombination breakpoints in region III for this genome and the rest of the genomes may indicate an increased recombination frequency at the end of the *pol* gene (position 4831 HXB2). Phylogenetic trees of regions III and V showed a separate location of this recombinant from other genomes from Russia, whereas in the region I tree, all samples clustered on one branch and, thus, the hypothesis of additional recombination is confirmed. However, because of the lack of available data, one can only speak of a unique recombinant form. Notably, the location of the recombination regions does not include the part of the genome that most often becomes the object of sequencing. Therefore, the prevalence of recombinants is likely underestimated and, thus, the rate of new recombination samples in Europe and Russia remains unclear. Only full-length genome sequencing of HIV-1 can help us understand the actual prevalence situation of these recombinant forms of the virus. Thus, the ratio of new recombination samples in Europe and Russia remains unclear.

In addition to subtype B, another subtype that is widely spreading in Portugal and in Spain is subtype G. The consequence of this was the emergence of several new circulating recombinant forms between subtypes B and G [19,20]. The last strain, CRF73, dates back to 1989. Ten years after this event, it is possible to speak about the emergence of another recombinant form, which was formed by subtype G strains from Spain and Portugal. The possible transmission of a new recombinant into the territory of Russia and its distribution dates back to the mid-2000s. The subsequent act of recombination between the new form and one of the strains of subtype B with the formation of a tertiary recombinant could already occur in Russia in 2014, as evidenced by the phylogenetic analysis.

The emergence of new recombinant forms is possible in the case of double infection with different subtypes. Recombination can lead to the appearance of chimeric molecules, which can later create a viral generation with different properties from the parents. The detection of such cases is a task for epidemiologists. The areas of greatest interest to physicians are the *pol* gene regions since it is this gene or rather its proteins, that account for the bulk of the existing drugs for HIV treating. However, as this study has shown, sequencing only these sites may not be sufficient for investigating viral population dynamics.

## 5. Conclusions

The near full-length genome sequencing of HIV-1 subtype G circulating in the Russian Federation was performed for the first time. This variant was isolated from patients infected during the nosocomial outbreak in 1988–1990 in the former USSR territory. The phylogenetic analysis results confirmed that the virus that caused the outbreak was related to the virus from the Democratic Republic of the Congo, which is in accordance with the literature data reporting the transmission of the virus from this region. Additionally, the near full-length genome sequencing of 8 samples that were determined as subtype G based on the *pol* region from patients identified in Russia in 2010-2015 was performed. The full genome sequence analysis, phylogenetic and recombination analyses showed that these samples are new recombinant forms of subtypes B and G. These samples are related to the virus from European countries, primarily Portugal and Spain. Recombination breakpoints, identified by jpHMM, do not include the protease and reverse transcriptase areas of the *pol* gene and, thus, cannot be subtyped properly when sequencing the *pol* region, which is routinely performed.

## Figures and Tables

**Table 1:** Characteristics of the Russian patients for whom near full-length genome sequences were obtained.

Patient ID	Sample	Gender	Route of Infection	Date of First Positive Immune Blot	Date of Collection	Accession Number
1	RU.1989.MF614605	male	nosocomial outbreak	1989	1989	MF614605
2a	RU.2012.MF614606	male	nosocomial outbreak	1990	2012	MF614606
2b	RU.2014.MF614607	male	nosocomial outbreak	1990	2014	MF614607
3	RU.2014.MF614608	male	unknown	2014	2014	MF614608
4	RU.2014.MF614609	male	unknown	2014	2014	MF614609
5	RU.2015.MF614610	male	sexual contact	unknown	2015	MF614610
6	RU.2015.MF614611	male	unknown	2010	2015	MF614611
7	RU.2015.MF614612	male	unknown	2014	2015	MF614612
8	RU.2016.MF614613	male	sexual contact	2010	2016	MF614613
9	RU.2016.MF614614	unknown	unknown	unknown	2016	MF614614
10	RU.2015.MF614615	male	unknown	2015	2015	MF614615
